# The research of knowledge diffusion network model for Tourism Destination-Public ecological civilization

**DOI:** 10.1371/journal.pone.0310112

**Published:** 2024-10-15

**Authors:** Jiehua Xu, Peng Peng, Dongping Wei, Zhijun Deng

**Affiliations:** 1 College of Architectural Engineering, Shenzhen Polytechnic University, Shenzhen, China; 2 College of Automobile and Communication, Shenzhen Polytechnic University, Shenzhen, China; 3 Institute of Applied Mathematics, Shenzhen Polytechnic University, Shenzhen, China; Zhejiang Gongshang University, CHINA

## Abstract

Visitor education plays a crucial role in the knowledge diffusion process in outdoor recreation and nature-based tourism. It entails sharing information, experiences, and insights with visitors to enhance their understanding and appreciation of the natural environment. Our methodology for investigating the diffusion of ecological civilization knowledge in tourism destinations involves constructing a knowledge diffusion network model. In this model, scenic spots, tourists, and the public are defined as network nodes, with the communication channels between them representing the edges of the network. By constructing a scale-free complex network, the knowledge diffusion mode of scenic spots can be depicted. The layer of resource supply node consists of different scenic spots, forming the core nodes set for knowledge diffusion in the tourism industry. This research aims to further explore the social and economic value of the tourism areas’ ecological civilization knowledge diffusion, as well as analyze the path, quantity, and cost of knowledge diffusion. by analyzing this knowledge diffusion network model, insights into the effectiveness and impact of visitor education in promoting ecological civilization and sustainable practices in tourism destinations can be gained. Overall, this approach provides a theoretical framework for investigating and comprehending the knowledge diffusion process in Tourism Destination-Public ecological civilization, thereby shedding light on the social and economic benefits that can be derived from sustainable tourism practices.

## Introductions

### Visitor education as knowledge service: Impact on socioeconomic development and tourism industry

Visitor education stands as a pivotal element of managing, conserving natural and cultural resources. Visitors can have a significant influence on the environment, especially in sensitive areas such as national parks, wildlife reserves, and historic sites. Educating visitors on responsible behavior can mitigate their impact, preserve the natural and cultural resources for future generations. Providing visitors with educational information about the resources they are visiting can enhance their experience and understanding of the place. This can lead to increased appreciation and support for conservation efforts. Some natural and cultural resources can be dangerous if not carefully handled or understood adequately. Educating visitors about safety guidelines and precautions can help prevent accidents and ensure a safe visit for everyone. Sustainable tourism involves meeting the needs of visitors while minimizing negative impacts on the environment, culture, and local economy. Educating visitors about sustainable practices can help promote responsible tourism and contribute to the long-term viability of the resource. Many resources bear cultural significance to local communities. Educating visitors about the cultural context of the resource can foster understanding and respect for local traditions and customs. Wei D. and Wen S. proposed that visitor education is essential to managing and conserving natural and cultural resources by promoting responsible behavior, enhancing experiences, ensuring safety, promoting sustainable tourism, and understanding cultural sensitivity.

In the era of knowledge economy, Davis C and Piva M proposed that knowledge replaces traditional resources such as capital, land and labor force and becomes the dominant resource element [1, 2]. As an informal learning pattern beyond traditional school education, tourism has an important influence on the overall layout of "Five-sphere integrated plan of China" consisting economic construction, political construction, cultural construction, social construction and ecological civilization construction. From the perspective of visitor education, the essence of visitor education activity is a process of knowledge service. The value of knowledge service is the core value of visitor education. The research conducted by Wen S. shows that visitor education activities effectively alter the knowledge, attitude and behavior of tourists. Thus, visitor education can reduce the environmental loss and management cost of tourism destinations [3]. Simultaneously, the dissemination of tourism information dissemination is not only a process of new knowledge generation and diffusion, but also a process of transforming knowledge into new visitor education program. The visitor education is to enhance the visitor experience by providing them with knowledge and understanding about the exhibits, displays, or natural environment they are visiting. Winter P observed through practice that some visitor education projects with plenty knowledge and good service level have become a new economic growth point for many tourism destinations and tour operators [4]. Some scholars such as Dwyer and Forsyth also believe that knowledge is a very special asset, the cost of knowledge learning and diffusion is far lower than the production cost because of its non-exclusiveness, so knowledge has its economies of scale [5, 6]. Therefore, the quantitative estimation of the knowledge service function of tourism resources on different scales has become the key factor to solve the relationship between tourist education activities and social and economic development. Therefore, the quantitative estimation of the knowledge service function of tourism resources on different scales has become the key factor to solve the relationship between tourist education activities and social and economic development. To further clarify the contributions of various authors, we have included a table summarizing their key contributions (see Table 1).

**Table 1:** Contributions of analyzed authors.

Author	Contribution
Wei D. & Wen S.	Emphasized the importance of visitor education in resource management.
Davis C. & Piva	Proposed knowledge as the dominant resource element in the knowledge economy.
Winter P	Highlighted the economic impact of effective visitor education projects.
Dwyer, L. & Forsyth	Discussed the economies of scale in knowledge learning and diffusion.

### Visitor education and knowledge diffusion: A network model for ecological civilization in tourism destinations

In this paper, "civilization" refers to the complex societies with developed urban centers, sophisticated cultures, advanced social organization, and established legal and political norms. "Ecological civilization" extends this concept, emphasizing a sustainable model where economic and social development are balanced with environmental stewardship. Within tourism destinations, ecological civilization manifests as the integration of sustainable practices, environmental ethics, and conservation efforts into the tourism industry, ensuring that development does not compromise the well-being of future generations or the natural ecosystems upon which they depend. Nagata and Shirayama put forward that in 2020 that the effect of knowledge diffusion on social and economic development and its scale economy have also attracted great attention from scholars at home and abroad [7]. Su et al. [8] highlighted several key attributes of knowledge: its non-excludability, the phenomenon of increasing marginal returns, and the fact that its transfer incurs certain costs and may result in intangible losses. Rosalba put forward that the establishment and interaction of knowledge networks of university research institutions, companies, governments and other organizations contribute to economic growth in 2003 [9]. Ghio et al. discussed the scale economy of knowledge innovation, put forward the concept of endogenous and spillover of knowledge innovation [10]. They established a model to analysis the market basis of knowledge innovation spillover and the optimal efficiency of knowledge innovation spillover and the scale of knowledge diffusion. In 2019, Cheng and Shiu studied the knowledge diffusion structure in the economy and society through the network structure [11]. Cheng considered the dynamic change of the knowledge diffusion over time. And he also discussed the source, size and characteristics of scale economy of knowledge diffusion. In 2020, Marques et al. demonstrated that analyzing the influencing factors of knowledge diffusion forms the basis of knowledge diffusion modeling [12]. They believed that institutional factors, knowledge confidentiality, distance, policy, capital and imbalance of ideas determine the effect of knowledge diffusion in a certain degree. The knowledge of ecological civilization is inherently non-exclusive. The more ecological civilization knowledge the public understand, the more obvious it will be for environmental protection and restoration. It is also in line with the characteristics of increasing marginal return of knowledge. Addressing the relationship of knowledge diffusion networks in other areas, we find that these networks often serve as a robust basis for interpreting the dynamics in our study. For instance, the establishment and interaction of knowledge networks among universities, companies, and governments contribute significantly to economic growth and innovation. This perspective is crucial for understanding how visitor education and knowledge diffusion in tourism can lead to sustainable development and ecological conservation. The working paper by Baggio, Cooper, McLeod and Scott in 2008 describes basic definitions and computational techniques, examines the effects of network topology on dynamic properties, and illustrates these concepts using a tourism destination, elba, italy, as a case study. So it is theoretically feasible to establish a knowledge diffusion network model to further study the Tourism Destination-Public ecological civilization knowledge diffusion path, diffusion quantity, diffusion cost, social and economic value.

## Methodology

The process of knowledge dissemination in tourist destinations is a typical example of knowledge diffusion, which typically begins at nodes with high levels of knowledge content and communication skills and spreads to nodes with lower levels of these attributes. In other words, the level of knowledge content and communication skills within a network determines the direction and strength of knowledge diffusion. This means that tourist destinations with knowledgeable and skilled individuals are more likely to be the starting point for knowledge diffusion, as they are better equipped to share their knowledge with others. Additionally, the strength of knowledge diffusion is also influenced by the level of communication skills within the network, as individuals with strong communication skills are better able to effectively transmit information to others. Overall, the process of knowledge dissemination in tourist destinations is multifaceted and influenced by variety of factors, including the level of knowledge content and communication skills within the network. In the past, it was usually spread from scenic spots to tourists, and then from tourists to the public. In the information age, it will also spread directly to the public through "virtual travel paths" such as websites and Weibo. If we regard scenic spots, tourists and public as the network nodes of knowledge diffusion and regard the information communication channels constructed between them as the edge of the network, then there are three levels of diffusion path: the first layer is the resource supply node layer, comprising different tourist destinations. The second layer is the tourist node layer composed of visitors who directly engage with the knowledge diffusion network. These visitors can be defined as Field visitors. The third layer is the public node layer composed of public. They are friends or families of the Field visitors. Or they visit the tourism scenic through the "virtual travel path ". In that case, they can be defined as virtual visitors. They are the indirect service object set of knowledge diffusion network. These arguments have been completely discussed in authors’ previous published papers and the empirical studies conducted by other scholars.

According to the Statistical Bulletin of Culture and Tourism Development of the Ministry of Culture and Tourism of the people’s Republic of China 2019, the number of all kinds of A level tourist attractions in China reached 12402 at the end of 2019. Different scenic spots can be regarded as the transmission nodes with different knowledge in the network. For example, the Imperial Palace of Beijing contains extensive historical, architectural and artistic knowledge. Meanwhile, Huangshan, Lushan, Huashan, Taishan and other scenic spots contain rich knowledge of natural landscape, historical culture and ecological civilization. Because these core nodes contain different ecological and historical knowledge, and their technology and management of knowledge dissemination are different. These core node including these five star scenic spots More motivation and social responsibility to implement the dissemination of knowledge in the background of China. And the effect of knowledge dissemination in these core nodes is different. So the nodes in the core nodes set also need to spread knowledge with each other.

The process of knowledge dissemination in tourist destinations exemplifies knowledge diffusion, where nodes with high levels of knowledge and communication skills initiate the spread to less equipped nodes. This dissemination, traditionally from scenic spots to tourists and then to the public, now also occurs through digital platforms. By conceptualizing scenic spots, tourists, and the public as network nodes and their communication channels as network edges, we can model the diffusion path in three layers. This study employs a scale-free network model to analyze the dynamics and implications of knowledge diffusion in Tourism Destinations, highlighting the multifaceted nature and critical factors influencing this process.

### Basic assumptions of knowledge diffusion network in scenic spots

The knowledge diffusion network refers to the interconnected system through which knowledge and information are shared, transferred, and spread among individuals, organizations, or communities. This network typically involves various channels, such as interpersonal communication, formal training programs, informal exchanges, and digital platforms. The network includes both formal structures, like organizational hierarchies and communication protocols, as well as informal relationships and communities of practice. The working paper by Baggio, Cooper analysis static structural characterization of the network and a dynamic analysis of the information diffusion process, with discussion of the outcomes and implications for destination management. Developed and inspired by Baggio, the knowledge diffusion network in scenic spots can be defined as *G*_*c*_(*t*) = (*V*_*C*_,*E*_*C*_). *V*_*C*_ is represented as a set of given core nodes (scenic spot network), that is, the set of network members. *E*_*C*_ is the edge of the connection tree between network nodes. *N*_*c*_ = |*V*_*C*_| represents the total number of nodes in the core nodes set in time *t*. *A*_*t*_ = (*a*_*ij*_)_*t*_ represents the connection matrix in time *t*. If the scenic spot node *i* and *j* form a connection tree, then *a*_*ij*_ = 1, otherwise *a*_*ij*_ = 0. The degree of node *i* refers to the number of connection trees which are directly connected to the node. The degree of node *i* can be calculated by $d_{i}=\sum_{j=1}^{N_{C}}a_{ij}$. The degree of node reflects the ability of individual node to spread knowledge outward, and also reflects the node’s intensity of possession of network resources.

As the number of visitors and the virtual tourist groups of scenic spots grows at all times, the number of people in contact with those tourists after returning to their places of residence with newly accepted knowledge also grows. The reception, digestion and dissemination of knowledge of different people is different. And the distribution of interpersonal communication is uneven. The state of connectivity in the network and the degree of nodes are different. Therefore, the knowledge dissemination of scenic spots-visitors-public has two characteristics: the node growth of scale-free network and the preferred connection. First, the diffusion of tourism knowledge is not carried out on all network nodes at the beginning. It begins to spread from some nodes firstly, such as from the 5A-level scenic spots to the surrounding areas of the scenic spots, and then gradually spreads to other scenic spots throughout the country. Second, the spread of tourism knowledge is often started by well-known scenic spots. Because well-known scenic spots undoubtedly have richer network resources. Theirs ecological civilization management experience is also richer because the state invested more related resources. The exemplary effect of famous scenic spots is especially obvious. Therefore, famous scenic spots have more opportunities to other scenic spots comparing with general scenic spots. Their degree will be particularly rich. In the other words, the distribution of scenic spots’ degree is not uniform and has the characteristics of power law distribution. The connecting trees between nodes are gradually formed. The connecting trees between scenic spots are often formed from part of the local scenic spots, then spread to the nodes of the whole network.

As the number of visitors and virtual tourist groups to scenic spots continues to grow, so does the number of people who engage with these tourists upon their return to their places of residence, armed with newly acquired knowledge. However, the reception, digestion, and dissemination of this knowledge varies among different individuals. Interpersonal communication is also unevenly distributed, with varying levels of connectivity within the network and different degrees of nodes. Consequently, the knowledge dissemination process involving scenic spots, visitors, and the public exhibits two key characteristics: the growth of scale-free network nodes and preferred connections.

Firstly, the diffusion of tourism knowledge does not occur simultaneously across all network nodes at the outset. Instead, it begins spreading from select nodes, such as 5A-level scenic spots, to the surrounding areas before gradually extending to other scenic spots throughout the country. Secondly, the spread of tourism knowledge often originates from well-known scenic spots due to their richer network resources and ecological civilization management experience. These famous landmarks have a more significant impact on other scenic spots compared to ordinary ones because they possess more extensive networks and state-backed resources. The exemplary effect of renowned scenic spots is particularly pronounced, leading them to have more opportunities to connect with other attractions than lesser-known ones. As a result, the distribution of scenic spots’ degrees is non-uniform and follows power law distribution patterns. Connecting trees between nodes gradually form, with local scenic spots often serving as the starting point for establishing links between various attractions before expanding to encompass the entire network.

### Topology of knowledge dissemination network for nodes in scenic spots

Through the aforementioned analysis, it becomes evident that the dissemination of knowledge within scenic spot nodes exhibits characteristics of a typical scale-free network. Therefore, a scale-free network model (BA model) is employed to depict the topology of the knowledge dissemination network in scenic spots. The Barabási-Albert (BA) model is a popular model for generating scale-free networks. It’s based on the idea that new nodes in a network preferentially attach to already well-connected nodes, leading to a few nodes with many connections and many nodes with only a few connections. To use the BA model to depict the topology of the knowledge dissemination network in scenic spots following the 7 steps including:

Step1.initialization: Start with a small number of nodes and edges.
Step 2. Adding New Nodes: At each step, add a new node to the network. This new node will connect to a randomly chosen existing node.
Step 3. Weighted Edges: Instead of connecting the new node to any old node, you can introduce a weighted mechanism.
Step 4. Repeat: Steps 2 and 3 are repeated for a certain number of iterations or until the desired number of nodes is reached.
Step 5. Scaling: To ensure that the resulting network has a power-law degree distribution.
Step 6. Visualization: Once you have generated your scale-free network using the BA model, you can visualize it using various tools and techniques. Nodes with many connections (hubs) will stand out, while others will be less connected.
Step 7. Comparison with Empirical Data: After generating the network, you can compare its properties (like degree distribution, clustering coefficient, etc.) with empirical data from real-world scenic spot knowledge dissemination networks. This will give you insights into how well the BA model captures the characteristics of the real-world network.

**Fig 1:**
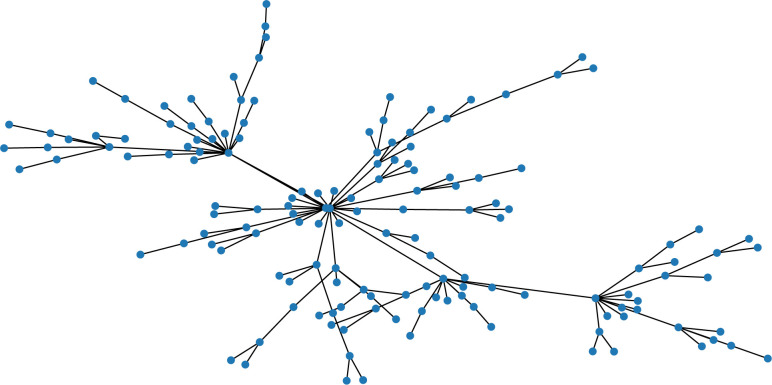
Scale-free network for knowledge dissemination in scenic spots.

At present, the methods of studying node degree distribution are as follows: mean field method [13, 14], rate-equation method [15], master equation method [16] and numerical calculation method based on Markov chain [17, 18]. The author uses the mean field method to study the distribution of scenic spots degree in knowledge dissemination network in this paper. Given that the scale-free network model differs from the actual situation of the knowledge dissemination network in the scenic area, the following algorithm is utilized to create a scale-free network, illustrated in Fig 1.

(1) Linear growth of nodes in scenic spots: the network of scenic spots starts with a small number of nodes *N*_*c*0_, adding a new node with *m*(*m*<*N*_*c*0_) edges within a certain time, and connecting the new nodes to the old nodes.

(2) preferential attachment: The probability that the new node is connected to the old node *i* depends on the degree of the node *i* and the attraction level of the scenic spot itself. Attraction level of the scenic spot is defined as *w*_*i*_. *w*_*i*_ is the abilities of the node attracting to other nodes, such as the ecological environment of the scenic spot, history and culture, management concept, tourist reputation, etc. Suppose the scenic spot has *n* attributions, it can be simply represented as $w_{i}=\frac{1}{n}\sum_{1}^{n}a_{i}$. The preference probability of the new node selecting the old node to connect is:

$$p_{i}=\frac{d_{i}+w_{i}}{\sum_{i}\left(d_{i}+w_{i}\right)}$$ (1)

In scenic area knowledge dissemination network, famous attractions often have a higher number of connections, which means they are easier to be visited and visited by tourists. Here are some of the reasons why the famous attractions are of a high level:

Popularity: Famous scenic spots often have high visibility, attracting a large number of tourists to visit. These attractions are usually publicized through various channels, such as media reports, travel guides, social media, etc., so that more tourists can know and choose to visit.

Convenient transportation: Many famous scenic spots are usually located in convenient places, such as city centers, railway stations, and near airports, which make it easier for tourists to reach these attractions. In addition, these attractions usually have good public transportation facilities, such as subway, bus, taxi, etc., to facilitate tourists to go to other scenic spots during the tour.

Rich tourism resources: Famous scenic spots usually have rich tourism resources, such as natural landscape, historical sites, culture and art, etc., which attract a large number of tourists to visit. At the same time, these scenic spots usually also provide a variety of tourism services, such as tour guides, accommodation, catering, etc., to further facilitate the tourists to visit.

Social network effects: The height of famous scenic spots is also influenced by social network effects. When more and more tourists choose to visit a famous scenic spot, the popularity and attraction of this scenic spot will be further increased, thus attracting more tourists. In addition, word-of-mouth among tourists will encourage more people to choose to visit these famous attractions.

Tourism policy and planning: The support and planning of the government and relevant departments will also affect the degree of famous scenic spots. For example, the government may invest in the infrastructure of tourist attractions to improve the accessibility and comfort of scenic spots, or formulate preferential policies to attract tourists to visit.

In short, the famous scenic spots have a high degree, which is the result of a combination of many factors. These famous scenic spots not only have high visibility and attraction, but also have good tourism resources and services, as well as convenient transportation conditions. Therefore, in the tourism network, these famous attractions are often the main destinations for tourists.

In the following section, it is an interesting way to demonstrate that the knowledge dissemination network of a scenic area can be theoretically considered as a scale-free network with power law distribution. To analyze the node distribution, it is useful to employ the mean-field method for calculation. The degree of the node in the network can be assumed to be *d*_*i*_. *d*_*i*_ is a continuous real value variable. According to the above two algorithms, the change rate of *d*_*i*_ is proportional to the probability *p*_*i*_. Thus the following differential equations are satisfied:

$$\frac{\partial d_{i}}{\partial t}=Ap_{i}$$ (2)

Taking into account *m* new nodes connect to an old node with degree *d*_*i*_ at a certain time interval, the increase degree of the network in time *t* is *A* = *m*. *m* can be obtained by using the following equation:

$$\frac{\partial k_{ij}}{\partial t}=m\frac{d_{i}+w_{i}}{\sum_{i}\left(d_{i}+w_{i}\right)}$$ (3)

where $\sum_{i}\left(d_{i}+w_{i}\right)=2mt+\sum_{i=1}^{t}w_{i}$, *w*_*i*_ obey a normal distribution with the mean value *μ* and the variance *σ*^2^. when *t* is sufficiently large, the following formula can be obtained.

$$\sum_{i}\left(d_{i}+w_{i}\right)\approx 2mt+\sum_{i=1}^{t}E(w_{i})=(2m+\mu )t$$ (4)

Substitute Eq (3) to Eq (4), the following equation can be derived.

$$\frac{\partial d_{i}}{\partial t}\approx m\frac{d_{i}+w_{i}}{(2m+\mu )t}$$ (5)

By solving differential Eq (5), the solution can be obtained.

$$\log(d_{i}+w_{i})=\frac{m}{\left(2m+\mu \right)}\log(t)+c$$ (6)

The initial condition of differential equation Eq (5) is as follows: *d*_*i*_(*t*_*i*_) = *m*.The solution of Eq (5) can be obtained as

$$d_{i}=(m+w_{i}){\left(\frac{t}{t_{i}}\right)}^{\frac{m}{2m+\mu }}$$ (7)

From Eq (7), it is evident that

$$p(d_{i}<d)=p((m+w_{i}){\left(\frac{t}{t_{i}}\right)}^{\frac{m}{2m+\mu }}<d)$$

$$=p(t_{i}>t{\left(\frac{m+w_{i}}{d}\right)}^{2+\frac{\mu }{m}})$$ (8)

Assuming the time interval of adding a new node is equal, then *t*_*i*_ is uniformly distributed

$$p(t_{i})=\frac{1}{N_{c0}+t}$$ (9)

When Eq (9) is substituted into Eq (8), the following can be obtained

$$p(d_{i}<d)=1-p(d_{i}>d)=1-\frac{t{\left(\frac{m+w_{i}}{d}\right)}^{2+\frac{\mu }{m}}}{N_{c0}+t}$$ (10)

The distribution of node’s degree in the constructed network by above algorithm can be calculated as follows

$$p(d)=\frac{\partial p(d_{i}<d)}{d}=\frac{t{\left(m+w_{i}\right)}^{2+\mu /m}}{N_{c0}+t}d^{-(3+u/m)}$$ (11)

The node degree distribution of scenic network is power law distribution with *γ* = 3+*μ*/*m*. According to the empirical research, the power index of network nodes in scenic spots is power law distribution with *γ* = 3, which is consistent with the theoretical model:

$$p(d)=\frac{2m^{2}t}{m_{0}+t}\frac{1}{d^{3}}$$ (12)

For example, Eq (3) shows that there are *N*_*c*0_ = 50 nodes initially, when adding a node in a certain time interval, then it can generate a scale-free network with 300 nodes show as following Fig 1.

The simulation in Fig 1 shows that as time increases, more non-core nodes are generated. This is because each new node generates a link to an existing node in the network, which can eventually form a scale-free network for Knowledge Dissemination in Scenic spots. The network’s structure is based on the Barabási-Albert (BA) model, where each new node has power law distribution with *γ* = 3 of being connected to the existing nodes in the network.

### Basic characteristics of scale-free network for knowledge diffusion in scenic spots

The clustering coefficient and the average path length reflect the two basic features of the network topology. The clustering coefficient reflects the degree of network node collectivization. In the social network, the group represents the circle of friends or acquaintances in the network. The members of the group are often familiar with each other and have a high degree of stability [19, 20]. There are also collectivization characteristics in the knowledge diffusion network of scenic spots. There are many close links in the same administrative region (the same province or city), so the clustering coefficient is also utilized to reflect the collectivization characteristics of the knowledge diffusion network of scenic spots. Clustering coefficient has different definitions [21]. The commonly used definitions are as follows: for a scenic spot node *i*, the set of nodes of directly connected scenic spots *i* is *V*_*i*_,then *n*_*i*_ = |*V*_*i*_|.The cluster coefficient of the scenic spot node can be defined as:

$$cl_{i}=\frac{\sum_{i,j\in V_{i}}a_{ij}}{\left(\begin{array}{c} n_{i}\\ 2 \end{array}\right)}$$ (13)

For all nodes in the scenic spots set, the average clustering coefficient is:

$$cl=\frac{1}{N_{c}}\sum_{i=1}^{N_{c}}cl_{i}$$ (14)

Network average path length represents how many steps a node takes on average to connect to another node, which reflects the effectiveness of communication between network nodes. s*l*_*ij*_ is the shortest path between scenic spot nodes *i* and nodes *j*, then the average path length of scenic spot network *L*_*c*_ is

$$L_{c}=\frac{1}{N_{c}(N_{c}-1)}\sum sl_{ij}$$ (15)

Robin Cowman think that the shorter network path length means faster knowledge diffusion, and the higher clustering coefficient can make the knowledge diffusion between the sub-groups of the network spread rapidly, but the exclusion of the sub-groups hinders the knowledge diffusion between the groups.

### Results

Under the above scale-free network topology, the dynamics of ecological knowledge dissemination network in scenic spots are further explored. The node of scenic spot can acquire ecological knowledge, historical and cultural knowledge and some management experience of how to spread knowledge through its own research, development and observation; on the other hand, the scenic spot can also acquire knowledge from other nodes by connecting trees. Wang et al. pointed out in 2015 that knowledge diffusion is affected by many factors, such as the learning ability of knowledge recipients, the reputation of knowledge communicators, and the communication situation between people [22]. The efficiency of knowledge diffusion tree between scenic spots is also affected by these similar factors. It is believed that the knowledge acquired by nodes in the connection tree is related to the knowledge content of the connected nodes, and is also influenced by the knowledge diffusion efficiency of the connection tree, as well as the number of connection trees.

After applying the proposed methods, we obtained several key results. Firstly, we observed that the knowledge content of each node increased over time, influenced by both the new knowledge generated internally and the knowledge acquired from connected nodes. Secondly, we found that nodes with a higher number of connection trees had a significantly greater capacity for knowledge diffusion, demonstrating the importance of establishing multiple connections within the network. Thirdly, the efficiency of knowledge diffusion varied across different connection trees, highlighting the role of communication channels and the learning abilities of the nodes involved.

Essentially, the knowledge contained in each node is different, so it can be reasonably assumed that each node in the core nodes set has varying abilities to spread knowledge. And the connection tree structure between nodes is different, so the efficiency of diffusion knowledge is also different. The total number of nodes in the core nodes set is *N*_*c*_ and the knowledge content of the node *i* in the core nodes set is *K*_c*node_i*_.According to the above assumptions, the knowledge content of the node *K*_*cnode_i*_(*t*) in time *t* should include the new knowledge *S*_*cnode_i*_(*t*) created by the node itself and the knowledge obtained by the connection tree from other nodes *K*_*ctree_i*_(*t*). *K*_*cnode_i*_(*t*) can be expressed by the following formula:

$$K_{cnode\_i}(t)=K_{cnode\_i}(S_{cnode\_i}(t),K_{ctree\_i}(t),n(K_{ctree\_i}(t)))$$ (16)

where $S_{cnode\_i}(t)=\sum_{0}^{t}S_{cnode\_i}(t)$, $K_{ctree\_i}(t)=\sum_{j=1}^{n(K_{ctree\_i}(t))}K_{ctree\_ij}(t)$. And *n*(*K*_*ctree_i*_) represent number of connection trees of node *i* at time *t*, then $n(K_{ctree\_i})=\sum_{j}a_{ij}$ and $0\leq n(K_{ctree\_i})\leq N_{c}-1$. It can be seen that the total knowledge storage of the whole core nodes set *K*_*cnode*_ at time *t* is:

$$K_{cnode}=\sum_{i=1}^{N_{c}}K_{cnode\_i}(t)=\sum_{i=1}^{N_{c}}K_{cnode\_i}(S_{cnode\_i}(t),K_{ctree\_ij}(t),n(K_{ctree\_i}(t)))$$ (17)

**Property 1:** the knowledge content *K*_*cnode_i*_(*t*) of the core node *i* should increase with the new knowledge generated by the node itself, that is $\frac{\partial K_{cnode\_i}}{\partial S_{cnode\_i}}>0$, with the increase of the knowledge obtained from the connection tree, that is $\frac{\partial K_{cnode\_i}}{\partial K_{ctree\_i}}>0$, with the increase of the number of connection trees of node *i*, that is $\frac{\partial K_{cnode\_i}}{\partial n(K_{ctree\_i})}>0$.

*K*_*cnode_i*_(*t*) can be represented by the following simple model by Property 1,

$$K_{cnode\_i}(t)=a_{ci}S_{cnode\_i}(t)K_{ctree\_i}(t)$$ (18)

From the previous hypothesis, it becomes evident that the structure of the connection tree between the two nodes varies, aligning more accurately with the actual scenario. The communication channels established by the two scenic spots are different, and the efficiency of communication is also different. The amount of knowledge of nodes *i* obtained by the connection tree between nodes *i* and *j* at time *t* is decided by the width of the communication path of the connection tree *w*_*cij*_, the flow rate of the communication path *v*_*cij*_, the knowledge content on the left side of the communication path *K*_*cnode_i*_, and the knowledge content on the right side of the communication path *K*_*cnode_j*_. Hence *K*_*ctree_ij*_(*t*) can be expressed as follows:

$$K_{ctree\_ij}(t)=K_{ctree\_ij}(w_{cij},v_{cij}(t),K_{cnodei},K_{nodej})$$ (19)

It is assumed that the width of the communication path does not change with time in lifetime of the connection tree. The flow rate increases with time in the initial stage of the connection tree, and the flow rate of the communication path decreases until it drops to 0 after some time of the connection tree begin, that is $\frac{\partial v_{cij}(t)}{\partial t}\geq 0$ and $\frac{\partial^{2}v_{cij}(t)}{\partial t^{2}}<0$. In addition, the knowledge content difference between the left and right sides of the communication path $\Delta K_{cnode\_ij}=K_{cnode\_j}-K_{cnode\_i}$, if Δ*K*_*cnode_ij*_≤0, then the flow rate of the communication path decreases to 0. The following indication function can be expressed

$$1_{\left(\Delta K_{cnode\_ij}\right)}=\{\begin{array}{l} 0\;\Delta K_{cnode\_ij}\leq 0\\ 1\;\Delta K_{cnode\_ij}>0 \end{array}$$ (20)

Eq (19) can be further expressed as follows

$$K_{ctree\_i}(t)=\sum_{j=1}^{n(K_{ctree\_i})}K_{ctree\_ij}(t)=\sum_{j=1}^{N_{c}}a_{ij}b_{cij}1_{\left(\Delta K_{cnode\_ij}\right)}w_{cij}v_{cij}(t,\Delta K_{cnode\_ij})$$ (21)

where *b*_*cij*_ represents the absorption coefficient of the node *i* in the process of knowledge diffusion from the node *j* to the node *i*. It represents the ability of the node *i* learning new knowledge. By substituting Eq (21) into Eq (18), the following can be derived:

$$K_{cnode\_i}(t)=a_{ci}S_{cnode\_i}(t)\sum_{j=1}^{N_{c}}a_{ij}b_{cij}1_{\left(\Delta K_{cnode\_ij}\right)}w_{cij}v_{cij}(t,\Delta K_{cnode\_ij})$$ (22)

The knowledge storage *K*_*cnode*_ the whole core nodes set in Eq (17) can be further expressed as

$$K_{cnode}=\sum_{i=1}^{N_{c}}K_{cnode\_i}(t)=\sum_{i=1}^{N_{c}}(a_{ci}S_{cnode\_i}(t)\sum_{j=1}^{N_{c}}a_{ij}b_{cij}1_{\left(\Delta K_{cnode\_ij}\right)}w_{cij}v_{cij}(t,\Delta K_{cnode\_ij}))$$ (23)

## Numerical simulation and empirical study

Because of the large number of scenic spots in the country, it’s difficult to do empirical research. The knowledge diffusion of Hainan tourist attractions is taken as an example for conducting the simulation analysis. According to the relevant information of the Department of Tourism, Culture, Radio, Television and Sports of Hainan Province, Hainan Province has a total of 66 A level scenic spots by 20 May 2020.There are 6 5-A level scenic spots, 20 4-A level scenic spots, 28 3-A level scenic spots, 12 2-A level scenic spot. The distribution of the scenic spots is not uniform, and is power law distribution. The connection tree between nodes is gradually formed. The connection tree between scenic spots is often formed from some local scenic spots, then extends to the nodes of the whole network. Therefore, it is assumed that the initial point of the network starts with three 5-A scenic spots. The tourism resources of knowledge, ecological management technology, ecological civilization and knowledge dissemination method of the three scenic spots can fully exchange or transfer through the connection tree between each other.

The initial connection matrix *A*_0_ that can be. A level scenic spot gradually establishes the connection tree with the 5-A scenic spot to communicate. It is assumed that one 4-A level scenic spot enters the network and establishes connections with two or three of the existing 5-A scenic spots after a certain period of time. The scenic spot try to establish communication channels for the dissemination of knowledge and technology with other scenic spots. The connection matrix of the scenic spot is *A*_1_.The following Table 2 is describing a network of scenic spots, where each spot has a certain level (e.g., 5-A), and they establish connections with each other over time to share knowledge and technology. The connection matrix would be a table that shows which scenic spots are connected to each other. For example: scenic spot A is connected to spots B and D. Scenic spot B is connected to spots A and C and E. Scenic spot C is connected to spots B and D. Scenic spot D is connected to spots A, C and E. Scenic spot E is connected to spot D.

**Table 2:** The initial connection matrix.

	A	B	C	D	E
A	-	1	0	1	0
B	1	-	1	0	0
C	0	1	-	1	0
D	1	0	1	-	1
E	0	0	0	1	-

If a new 4-A level scenic spot enters the network and establishes connections with two or three of the existing 5-A scenic spots, you would update the connection matrix accordingly. For example:

In this updated matrix, the new 4-A level scenic spot F is connected to spot E shown in Table 3.

**Table 3:** The connection matrix.

	A	B	C	D	E	F
A	-	1	0	1	0	-
B	1	-	1	0	0	-
C	0	1	-	1	0	-
D	1	0	1	-	1	-
E	0	0	0	1	-	-
F	-	-	-	-	-	1

From the previous theory, it is evident that the probability of the new node choosing whether to connect to the old node depends not only on the degree of the node, but also on the attraction degree of the scenic spot itself. After the period *t* = 40, a scale-free scenic spot network with power law distribution is formed, and the complete connection matrix is *A*_*t*_. In this network framework, it can be calculated the total amount and efficiency of network knowledge dissemination.

Different scenic spots have different information, such as historical and cultural information, human and geographical information, natural environment information, and scenic spots ecological civilization management information, dissemination of knowledge and experience information. Some of these information can serve tourists and the public, and the information such as the management experience of scenic spots, the abilities of knowledge dissemination can be exchanged between managers of these scenic in the network. Different types of A level scenic spots contain different amounts of information. Generally speaking, it is considered that 5A level scenic spots contain the greatest amount of information. 2A level scenic spots contain the smallest amount of information. Assuming that 5A level scenic spots contain between 40,000 and 50,000 pieces of information. 4-A level scenic spots contain between 30,000 and 40,000 pieces of information.3-A level scenic spots contain between 20,000 and 30,000 pieces of information. 2-A level scenic spots contain between 10,000 and 20,000 pieces of information. Uniform distribution information data for 40 scenic spots is generated randomly using a MATLAB program. In the data set generate by program, 5-A level scenic spots have an average of 2.09 million pieces of information. 4-A level scenic spot has 3.12 million messages.3A level scenic spots have 270.77 million messages, 2A level scenic spots have 1051 2 million messages. The total amount of knowledge of all nodes in the initial time is (10,000):

$$K_{cnode}(0)=\sum_{i=1}^{N_{c}}K_{cnode\_i}(0)=115.37$$ (24)

The width of the communication path does not change with time. The flow rate increases with time in the initial stage of the connection tree, and the flow rate of the communication path decreases until it drops to 0 after some time. In addition, the knowledge content difference between the left and right sides of the communication path, then the flow rate of the communication path decreases 0.Under these hypothesis, the total amount of knowledge dissemination in the network after *t* = 40:

$$K_{ctree\_i}(40)=\sum_{j=1}^{n(K_{ctree\_i})}K_{ctree\_ij}(40)=73.08$$ (25)

The knowledge storage of the whole core nodes set at time *t* = 40 is *K*_*cnode*_ (ten thousand):

$$K_{cnode}(40)=\sum_{i=1}^{N_{c}}K_{cnode\_i}(40)=188.45$$ (26)

The average knowledge content of all nodes is (10,000):

$$average(K_{cnode})=\sum_{i=1}^{N_{c}}K_{cnode\_i}(40)/N_{c}=5.1683$$ (27)

In the ecological civilization knowledge dissemination network in the core node layer, the knowledge storage in the core nodes is 1.6334 times than the beginning. This indicates that a rapid augmentation of knowledge storage in the core nodes facilitated by knowledge dissemination in the network. The average knowledge content among all scenic spots greatly increase after a period time. This means that as more users are connected to the network, the total amount of knowledge stored in the core nodes increases rapidly. This could lead to a significant increase in the diversity and richness of knowledge available for users within the network. After a period of time, the average knowledge content among all scenic spots greatly increases. This could be due to several factors such as more people being connected to the network, new information being added to the network, or changes in user behavior that contribute to a greater number of new knowledge submissions.

## Discussion

The result in this paper appears that the ecological civilization knowledge dissemination network has been effective in increasing the knowledge storage within its core nodes. This rapid increase in knowledge storage can be attributed to the network’s ability to disseminate information efficiently among its members.

Several studies have highlighted the importance of knowledge dissemination networks in promoting sustainable development and environmental conservation [23, 24]. These networks often consist of various stakeholders, including government agencies, non-governmental organizations (NGOs), and local communities, who collaborate to share information and resources related to ecological issues [25]. This aligns with our findings, demonstrating the critical role of such networks in enhancing knowledge storage and dissemination within an ecological context.

One key factor contributing to the success of these networks is their ability to establish core nodes or hubs that serve as central points for knowledge exchange [26]. These core nodes often have more resources and expertise than other members of the network, which allows them to play a critical role in facilitating knowledge dissemination [27]. In our study, the effectiveness of core nodes as hubs for knowledge dissemination is evident, supporting previous research that emphasizes their importance in knowledge networks.

In this study, the observed increase in knowledge storage within the core nodes suggests that these nodes are effectively serving as hubs for ecological civilization knowledge dissemination. This finding aligns with previous research that has shown that well-connected core nodes can enhance the overall efficiency and effectiveness of knowledge networks [28]. The increase in knowledge storage within core nodes highlights their pivotal role in the network, reinforcing the idea that strong central nodes are essential for efficient knowledge diffusion.

Moreover, the observed increase in the average knowledge content among all scenic spots indicates that the network is fostering a culture of learning and innovation within the ecological civilization community. This is an important outcome, as it suggests that the network is not only benefiting its core nodes but also extending its positive impacts to other members of the ecosystem. This broader increase in knowledge content across the network underscores its comprehensive impact, promoting a widespread culture of knowledge and innovation.

In conclusion, our results provide valuable insights into the effectiveness of ecological civilization knowledge dissemination networks in promoting sustainable development and environmental conservation. By establishing core nodes and facilitating knowledge exchange among its members, these networks can help bridge the gap between theory and practice in ecological governance and contribute to a more resilient and sustainable future for all. These findings highlight the practical applicability of our results, demonstrating how well-structured knowledge networks can support ecological governance and sustainable development on a broader scale.

## Conclusions and prospects

In conclusion, the study reveals that the dissemination of ecological civilization knowledge within scenic spots is contingent upon core nodes. These nodes command influence through their credibility and their ability to effectively impart knowledge about natural landscapes, history, and culture. Their extensive connections are conduits for rapid information transmission and the adoption of sustainable practices.

A scale-free network model was utilized to elucidate the structure of core nodes within knowledge dissemination networks. The model highlights the pivotal role of hubs in the network, crucial for the spread of ecological civilization knowledge. Analysis of network metrics, such as node degree distribution and clustering coefficients, has led to the identification of key nodes that substantially influence the network’s flow of knowledge.

The study’s objective was to understand the role and structure of core nodes in the dissemination of ecological knowledge. The study also uncovers an uneven distribution of connections among scenic spots, with certain spots emerging as significant hubs. This distribution pattern proposes that a strategic emphasis on these hubs could enhance the efficiency of knowledge dissemination throughout the network. Empirical outcomes from Hainan Province indicate significant enhancements in knowledge retention at these core nodes, evidenced by a 1.6334-fold increase in knowledge storage among the top-rated scenic spots. This surge is a clear indication of the success of educational endeavors in these regions.

However, the research acknowledges its limitations, particularly its concentrated focus on the core node layer. Subsequent inquiries should examine the interactions between core and peripheral nodes. Further, the integration of tangible data, including visitor behavior and environmental factors, could yield a more intricate understanding of the patterns of knowledge dissemination. Prospective research paths involve an in-depth exploration of the peripheral nodes’ roles, the influence of seasonal and demographic variables, and the inclusion of actual events into the analytical models. These future directions will deepen the comprehension of ecological civilization knowledge networks and support the enhancement of educational and sustainable tourism practices.

In summary, the study achieved its objective by identifying the structure and significance of core nodes in knowledge dissemination networks. The results underscore the relevance of these nodes in enhancing knowledge retention and promoting sustainable practices within scenic spots. Future research focusing on peripheral nodes and additional variables will further refine these insights, aiding the development of more effective knowledge networks and sustainable tourism strategies.

## Supporting information

S1 Data (XLSX)
